# A Waveguide Inline Binary Metasurface for Wavelength-Selective Transmission and Standing Wave Focusing

**DOI:** 10.3390/nano14040367

**Published:** 2024-02-16

**Authors:** Chun-Hyung Cho, Hyuntai Kim

**Affiliations:** Department of Electronic and Electrical Converged Engineering, Hongik University, Sejong 30016, Republic of Korea; chcho@hongik.ac.kr

**Keywords:** metasurface, hollow-core fiber, inline metamaterial, binary lens, standing wave focusing

## Abstract

This study presents an innovative inline metasurface design for selective wavelength transmission and focusing. When integrated into optical fibers, it improves the stability and compatibility with techniques like wavelength division multiplexing and phase modulation. Precise parameters, determined through analytical calculations and simulations, allow for the design of multifunctional lenses within the optical fiber platform. The numerical results demonstrate unmodulated transmission for specific wavelengths, while others exhibit standing wave focusing with a 0.67 μm beam radius and a 0.31 μm depth of focus. This technology holds promise for applications in quantum experiments, sensing, and optical communication.

## 1. Introduction

Optical focusing has a long history of research and versatile applications across various fields [1,2,3]. In recent times, it has gained prominence in imaging, communication, sensing, precision machining, trapping, and notably in quantum mechanics research, facilitating experiments involving cold atoms [4,5,6,7,8,9]. In the realm of optical trapping, minimizing the depth of focus is of paramount importance. To achieve this, a standing wave focusing approach, achieved by converging light from both directions, is commonly employed [10,11,12]. However, this technique necessitates precise control of positions and phase adjustments.

Concurrently, advancements in fabrication and measurement technologies have enabled the production of materials on the nanoscale, allowing for the observation of phenomena at this minuscule level [2,13,14]. In optics, the advent of nanophotonics has given rise to developments in thin optics, plasmonics, and metamaterials [15,16,17,18,19,20]. Fabricating structures smaller than the wavelength of light results in the medium appearing as a homogeneous substance from the perspective of light, affording engineers with the ability to manipulate its properties. The emergence of metamaterials has spurred research into novel thin-film lenses and multifunctional metamaterials that exhibit varying behaviors contingent on the incident polarization and wavelength. Multifunctional lenses, for instance, can focus light of specific wavelengths while allowing others to transmit unaltered, making them highly suitable for applications in quantum mechanics experiments and trapping [21,22,23].

This study introduces an innovative inline metasurface structure that facilitates the transmission of specific wavelengths while concurrently enabling the focusing of others. Existing free-space metamaterial setups encounter challenges related to alignment and efficiency, as the device size has been shown to be relatively small in comparison to the beam size. Additionally, achieving compatibility with input/output equipment poses further difficulties. Recent research has explored enhancing the efficiency by attaching metasurfaces to the cross-section of solid-core optical fibers and connecting them to hollow-core fibers to enhance the stability and convenience [24,25]. Within this framework, multifunctional lenses will be fabricated on both ends of solid-core fibers and subsequently interconnected via hollow-core fibers. The use of optical fibers as a platform not only simplifies the introduction of two different wavelengths using modalities such as wavelength division multiplexing (WDM), but also facilitates phase modulation using phase modulators and similar devices. Importantly, once the length of the hollow core fiber is determined, there is no longer a need for distance adjustments between the two lenses. The fabrication of multifunctional lenses employs a binary dielectric structure, with adjustments in the wavelength and thickness to confer identical phase properties to one wavelength and opposite phase properties to the other. This study has innovatively presented diverse multifunctional designs tailored to different wavelengths, accomplished through a streamlined process and the utilization of a singular material. Additionally, the introduction of a fiber-optic platform metamaterial ensures heightened efficiency, stability, and versatility. Furthermore, the proposal of a fiberized schematic with a variable focal position introduces a novel experimental dimension. These innovative attributes signify the distinctiveness of our methodology, providing a unique perspective compared to conventional approaches.

## 2. Materials and Methods

**Figure 1:**
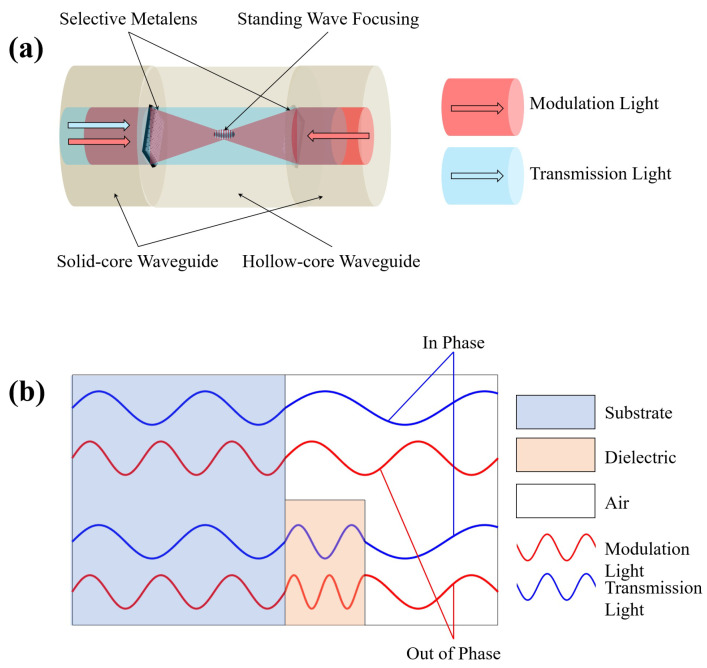
(**a**) Schematic of the proposed inline selective focusing metasurface. (**b**) The design principle of the selective metamaterial block.

The proposed configuration entails the incorporation of selective metamaterials onto the facets of both solid-core waveguides, with subsequent interconnections using a hollow-core waveguide. In this design, the transmission light is permitted to propagate without any modulation, while the modulation light is directed towards a focal point. Additionally, to optimize the efficiency of the focusing and reduce the depth of focus, the introduction of modulation light in both sides is considered, achieving standing wave focusing. This groundbreaking system offers several notable advantages when employing optical fibers or alternative waveguides, including the facile manipulation and adjustment of two light wavelengths through the use of components such as WDM and phase modulators. A comprehensive conceptual diagram of the system is depicted in Figure 1a.

The structure of the selective metasurface is straightforward. By introducing a dielectric block, we select the thickness and wavelength corresponding to the transmission light, which imparts a phase change of 2mπ, and for the modulation light, the phase change is set to (2m−1)π. As a result, the transmission light proceeds, unaffected by the presence of the dielectric block, while, from the vantage point of the modulation light, the dielectric block operates analogously to a zone plate. This configuration can be applied not only to focusing plates but also to other structures, including multifocusing, superoscillatory lenses, and in diffraction. The underlying principle of this structure is depicted in Figure 1b. As shown in the figure, the red modulation light exhibits an out-of-phase condition, with opposite phases, when compared to the scenarios with and without the dielectric present. Conversely, the blue transmission light satisfies the in-phase condition, irrespective of the presence of the dielectric, maintaining the same phase.

The selection process for the block thickness and wavelength is as follows. We assume the substrate to be silica [26] and the block to be silicon [27]. We apply a transmission wavelength of 1550 nm, commonly used in communication. After calculating the thickness that leads to an in-phase change at 1550 nm, we identify the wavelength at which an inversive phase change (out-of-phase condition) occurs at that specific thickness. These computations are performed using COMSOL Multiphysics, a numerical simulation tool. Specifically, we employed an optical module based on the finite element method.

Upon finalizing the specifications for the block and the wavelength, the subsequent step involves determining the length of the hollow-core waveguide before proceeding to construction of the zone plate. Given that the hollow region effectively functions as a cavity, the extent of this region will influence the transmission efficiency, especially in the context of standing wave focusing. Consequently, it is imperative to set the cavity length as a primary consideration before fine-tuning the focal length to precisely converge the light at the central point. Following the establishment of the cavity length, the fabrication of a binary zone plate is achieved through the utilization of the block. The design methodology employed for the zone plate centers around the inversive design technique, known as the virtual point source method [28].

## 3. Results

Initial analytic calculations were carried out to establish the conditions for a phase change of 2mπ within the silicon block and air for the transmission light with a wavelength of 1550 nm. The pertinent expression is presented in Equation (1),

(1) $$k_{t,si}d-k_{t,air}d=\frac{2\pi }{\lambda_{t}}n_{si}d-\frac{2\pi }{\lambda_{t}}d=2m\pi ,\left(m=1,2,\ldots \right)$$

where $k_{t,material}$ denotes the wavenumber of the transmission light in a certain material, $n_{si}$ denotes the refractive index of silicon, $\lambda_{t}$ signifies the wavelength of the transmitted light, and *d* represents the thickness of the block. The outcome of the calculations reveals that the value of *d* must be a multiple of 625 nm to satisfy the stated condition. For the sake of simplicity, in the analysis, the case with the smallest integer value of *m* is considers, which is m=1. Subsequently, the formula in Equation (2) is employed to compute the wavelength of the modulation light at the specific thickness mentioned earlier.

(2) $$k_{m,si}d-k_{m,air}d=\frac{2\pi }{\lambda_{m}}n_{si}d-\frac{2\pi }{\lambda_{m}}d=\left(2m-1\right)\pi ,\left(m=1,2,\ldots \right)$$

where $k_{m,material}$ denotes the wavenumber of the modulation light in a certain material and $\lambda_{m}$ signifies the wavelength of the modulation light. Note that the term kd in Equations (1) and (2) signifies the spatial variation of the phase. Assuming the refractive indexes are identical, the feasible wavelength options are 3100 nm, 1033.3 nm, 620 nm, and so forth, which are $2\lambda_{t}/\left(2m-1\right)$. Among these choices, 1000 nm is deemed optimal due to its ease of generation using Ytterbium lasers, its ready availability in commercial settings, and its minimal deviation from 1550 nm, thereby rendering it compatible with the same waveguide architecture.

However, it is imperative to consider the effects of material dispersion in practical scenarios. Moreover, as light propagates through the three regions of substrate, block and air, reflection at each interface must also be taken into account. Therefore, to obtain more accurate results, numerical simulations were conducted under these specific conditions.

**Figure 2:**
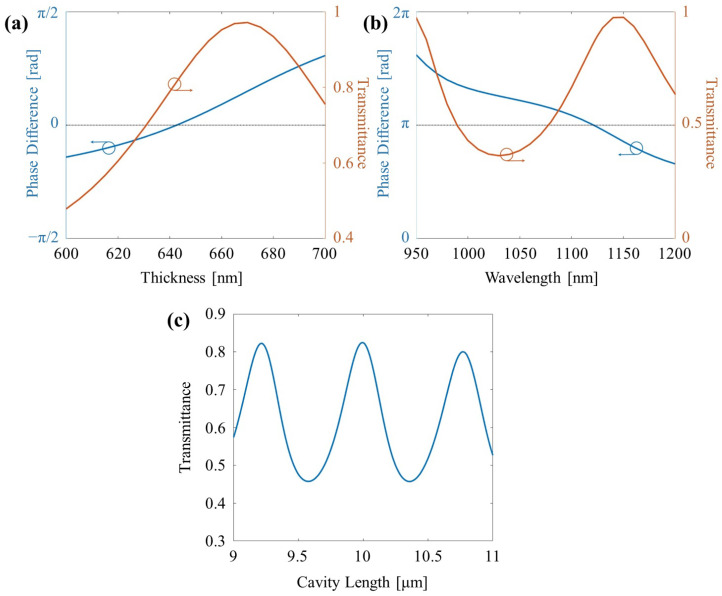
(**a**) Phase difference and transmittance of the transmission light in terms of the dielectric thickness. (**b**) Phase difference and transmittance of the modulation light in terms of wavelength. (**c**) Transmittance of the transmission light in terms of the cavity length.

Figure 2a presents the phase change difference and transmittance as a function of the block thickness at the transmission wavelength. The phase change refers to the phase difference between the presence and absence of the block, which can be expressed as $k_{t,si}d-k_{t,air}d$, and the transmittance represents the transmission through the silicon block. A sweep was conducted around the theoretically calculated value of 625 nm. The computational results show that at 642.6 nm, the transmission with the block present is 81.4%, slightly lower than the 97.86% without the block. Nevertheless, a minimal phase difference of −0.00031 rad was observed.

Figure 2b illustrates the phase change difference and transmittance through silicon as the wavelength is varied around 1033.3 nm, which corresponds to the previously calculated block thickness of 642.6 nm. A sweep was conducted in the vicinity of the theoretical approximation. The computational results reveal that at 1121.2 nm, the transmittance is 86.27%, and the phase difference is only −6.17 $\times \;10^{-5}$ rad with respect to π.

Subsequently, the cavity length is established, representing the distance spanning from the left lens surface to the right lens surface. The lenses are fashioned as arbitrary Fresnel zone plates, which focus light by alternately filling and leaving empty concentric rings [24,29,30], combining the silicon block and air. The intended design seeks to achieve a focal point of approximately 5 μm, necessitating a sweep of the cavity length in the vicinity of 10 μm. The transmission of 1550 nm Gaussian light with a beam radius of 6 μm as a function of the cavity length is illustrated in Figure 2c. The results demonstrate that, as anticipated, local maxima are observed at half-wavelength intervals of 1550 nm. Among these, a local maximum closest to the targeted value of 10 μm is identified at 9.99 μm, which will be employed for subsequent analyses. At this particular cavity length, the calculated transmittance is determined to be 82.44%.

**Figure 3:**
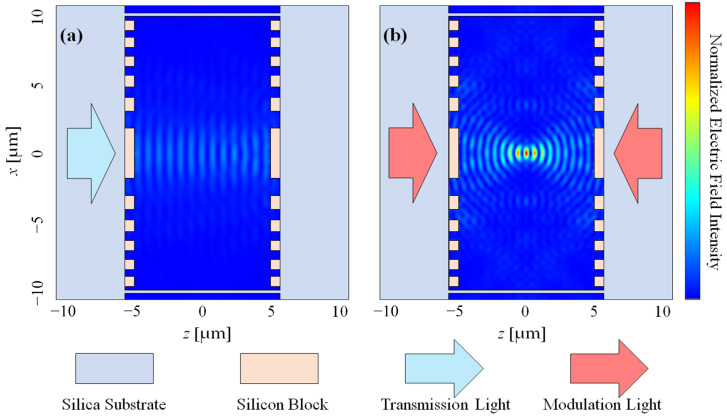
Calculated normalized electric field intensity of (**a**) transmission light and (**b**) modulation light, respectively.

Utilizing the derived parameters for the silicon block and the hollow core cavity, the lens was designed, with the focal length precisely set at half of the cavity length, i.e., 4.995 μm. Visualizations of the electric field profiles of the transmission light incident from one side and the modulation light incident from both sides are presented in the ensuing figure. Figure 3a depicts the field pattern of the transmission light when introduced from one side. Although the presence of Fresnel reflection components originating from the opposing side gives rise to a fringe pattern akin to a standing wave, it is evident that the Gaussian beam remains undisturbed and propagates linearly. Conversely, in Figure 3b, the modulation light is visibly centralized due to the selective lens. In this scenario, the bidirectional modulation light leads to the observation of a fringe pattern of focused light in the central region as well. The full width at half maximum (FWHM) of the beam size was also calculated. The FWHM of the beam radius (*x* axis) was calculated as 0.67 μm, and the FWHM of the depth of focus (*z* axis) was calculated as 0.31 μm.

## 4. Discussion

**Figure 4:**
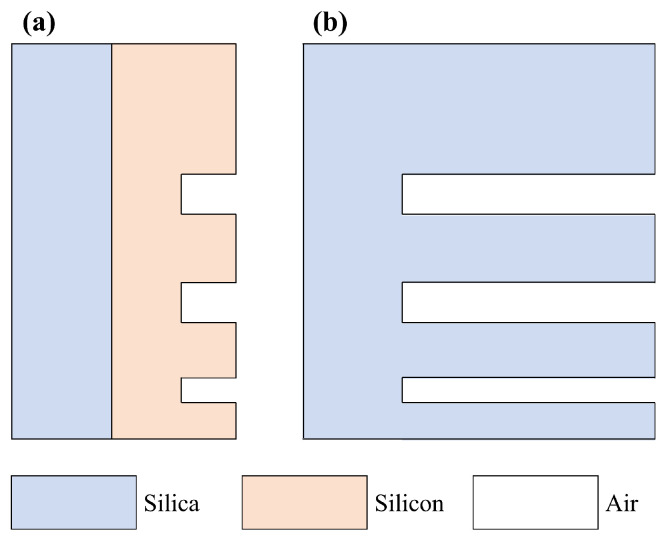
(**a**) Model for the case where silicon is not completely etched from all areas. (**b**) A model consisting of a single material (silica).

While the impact of the presence or absence of the block on phase modulation is minimal, thereby circumventing significant phase front distortions, divergences in transmission levels can potentially induce alterations in the intensity distribution. To mitigate this, two distinct strategies can be used. Firstly, as depicted in Figure 4a, an additional layer of silicon could be deposited onto the substrate, inducing an intentional reduction in the overall transmission. This approach retains the desired intensity distribution at the expense of overall efficiency.

Conversely, an alternative methodology, exemplified in Figure 4b, entails employing uniform materials in both the substrate and the block. This entails patterning the silica substrate directly to establish the desired structure. Nevertheless, due to the relatively modest refractive index difference between silica and air, this technique necessitates considerably elevated block heights. Upon analytical calculations guided by Equation (1), it becomes evident that adherence to the in-phase condition necessitates a block thickness of no less than 3.49 μm concerning the 1550 nm transmission wavelength. Such an approach not only accentuates fabrication intricacies but also jeopardizes the structural robustness. Furthermore, the potential emergence of unanticipated surface effects necessitates cautious consideration.

**Figure 5:**
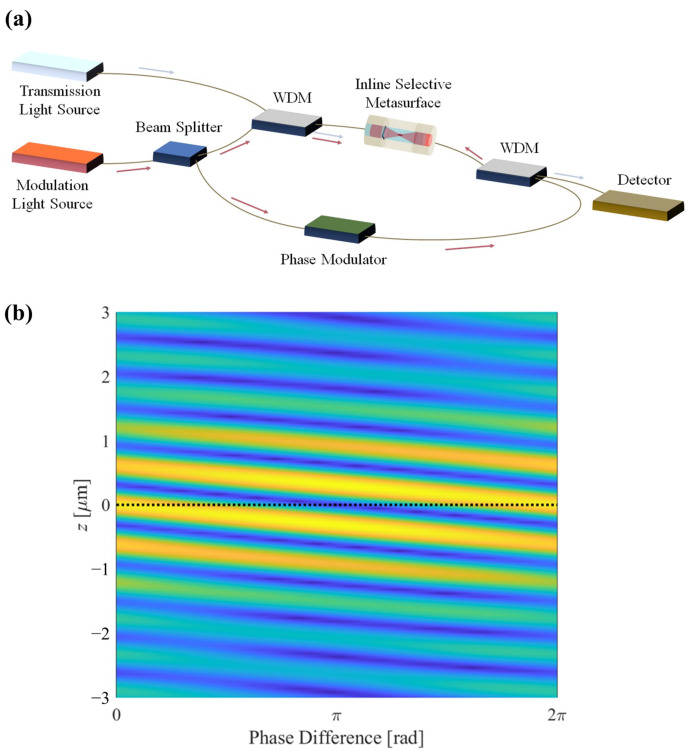
Calculated electric field intensity of the liquid-crystal-filled optical component. The red arrows show the electric field polarization of x-axis and y-axis.

Furthermore, in the context of bidirectional focusing, achieving precise phase alignment is challenging. Therefore, employing a phase modulator in practical experimentation proves to be advantageous. Consequently, the overall schematic configuration resembles that illustrated in Figure 5a. Under such circumstances, the electric field magnitude profile resulting from altering the phase on one side is depicted in Figure 5b. Notably, with a change in phase, an effect becomes evident where the fringe pattern of the focal point undergoes displacement, thereby enabling fine-tuning of the focal point through phase modulation. This feature holds the potential for subtle adjustments in the position of trapped particles, facilitating minute spatial displacements.

## 5. Conclusions

To conclude, we have introduced a novel approach to optical standing wave focusing using an inline metasurface structure, offering selective transmission and modulation of specific wavelengths. By applying this concept to solid-core and hollow-core optical fibers, we have demonstrated an enhanced efficiency, stability, and adaptability for various experimental setups.

Analytical calculations and simulations have enabled us to determine the optimal parameters for the silicon block, cavity length, and wavelengths. These parameters were used to fabricate multifunctional lenses, which, when incorporated into the optical fiber platform, provide precise control over two different wavelengths through WDM and phase modulation. Importantly, the determination of the hollow-core fiber’s length eliminates the need for subsequent distance adjustments between the lenses. The numerical results demonstrate unmodulated transmission for specific wavelengths, while others exhibit standing wave focusing with a 0.67 μm beam radius and a 0.31 μm depth of focus.

Furthermore, our results showcase the potential of achieving fine spatial displacements of the focal position through phase modulation, which can have significant implications for trapping and manipulating particles in quantum-related experiments. In summary, our innovative approach not only enhances optical trapping techniques but also holds promise for a wide range of applications in sensing, quantum mechanics research, and beyond.

## Data Availability

The data underlying the results presented in this paper are not publicly available at this time but may be obtained from the authors upon reasonable request.
